# An Endophytic Strain of *Bacillus amyloliquefaciens* Suppresses *Fusarium oxysporum* Infection of Chinese Wolfberry by Altering Its Rhizosphere Bacterial Community

**DOI:** 10.3389/fmicb.2021.782523

**Published:** 2022-01-05

**Authors:** Constantine Uwaremwe, Liang Yue, Yun Wang, Yuan Tian, Xia Zhao, Yang Liu, Qin Zhou, Yubao Zhang, Ruoyu Wang

**Affiliations:** ^1^Gaolan Station of Agricultural and Ecological Experiment, Northwest Institute of Eco-Environment and Resources, Chinese Academy of Sciences (CAS), Lanzhou, China; ^2^CAS Key Laboratory of Tropical Forest Ecology, Xishuangbanna Tropical Botanical Garden, Chinese Academy of Sciences (CAS), Mengla, China; ^3^University of Chinese Academy of Sciences, Beijing, China; ^4^Key Laboratory of Desert and Desertification, Northwest Institute of Eco-Environment and Resources, Chinese Academy of Sciences (CAS), Lanzhou, China

**Keywords:** *Bacillus*, biocontrol, *F. oxysporum*, root rot, wolfberry, rhizosphere bacterial community

## Abstract

Root rot disease is a serious infection leading to production loss of Chinese wolfberry (*Lycium barbarum*). This study tested the potential for two bacterial biological control agents, *Bacillus amyloliquefaciens* HSB1 and FZB42, against five fungal pathogens that frequently cause root rot in Chinese wolfberry. Both HSB1 and FZB42 were found to inhibit fungal mycelial growth, *in vitro* and *in planta*, as well as to promote the growth of wolfberry seedlings. In fact, a biocontrol experiment showed efficiency of 100% with at least one treatment involving each biocontrol strain against *Fusarium oxysporum*. Metagenomic sequencing was used to assess bacterial community shifts in the wolfberry rhizosphere upon introduction of each biocontrol strain. Results showed that HSB1 and FZB42 differentially altered the abundances of different taxa present and positively influenced various functions of inherent wolfberry rhizosphere bacteria. This study highlights the application of biocontrol method in the suppression of fungal pathogens that cause root rot disease in wolfberry, which is useful for agricultural extension agents and commercial growers.

## Introduction

Chinese wolfberry (*Lycium barbarum*) is a deciduous perennial plant of economic importance that grows well in the northwest, arid regions of China due to its salt tolerance, drought resistance, and fast-growing qualities (Byambasuren et al., 2019; Wang et al., 2019b). In China, wolfberry is used in traditional medicine because of its high content in bioactive secondary metabolites and its multitude of benefits to human health (Bucheli et al., 2011; Byambasuren et al., 2019; Wang et al., 2019a). Currently, the total planting area of wolfberry plants in China represents more than 1.33 × 10^5^ ha (Meng et al., 2019; Wang et al., 2019a). Unfortunately, wolfberry yield has been severely impacted by root rot, one of the most widespread and destructive soil borne diseases. In our previous study, we investigated root rot disease in Chinese wolfberry. *Fusarium* species were the most abundant among all isolated fungal pathogens, and *Fusarium* infected plants were characterized by yellow leaves, necrosis, death and rotten roots (Uwaremwe et al., 2021). It can be difficult to identify, measure and manage root rot disease in a nursery setting because pathogens may easily and quickly spread from plant to plant causing widespread death of seedlings (Omukhua and Godwin-Egein, 2011). Various fungicides are known to be effective against soil borne disease (Dhahira-Beevi and Qadri, 2010). However, an increasing use of chemical treatments causes several negative effects such as environmental pollution, imbalance in the soil ecosystem, potential threat to silkworms, and development of pathogen resistance (Compant et al., 2005; O’Brien, 2017). Thus, biocontrol using antagonistic microorganisms is a safer alternative to reduce the use of chemicals in agriculture, and it is considered as a promising approach for the management of soil borne diseases (Dhahira-Beevi and Qadri, 2010; Shahid and Khan, 2016; Singh et al., 2017; Alamri et al., 2019). Biocontrol Agents (BCAs) are potentially beneficial microorganisms including fungi, viruses and a group of bacteria called plant growth promoting rhizobacteria (PGPR) (Pal and McSpadden, 2006; Mota et al., 2017; O’Brien, 2017; Köhl et al., 2019). Members of *Bacillus* spp. and *Pseudomonas* spp. (bacteria), and *Trichoderma* spp. (fungi), have demonstrated abilities to suppress several soil borne plant pathogens, including species of *Streptomyces* and *Fusarium*, while also promoting plant growth (Singhai et al., 2011; Meng et al., 2013; Saravanakumar et al., 2017). Thus, they can serve simultaneously as both biopesticide and biofertilizer. PGPR colonize the root surface and the closely adhering soil interface (i.e., the rhizosphere) and some of them can also enter the root interior as endophytes (Compant et al., 2005; Beneduzi et al., 2012; Lyu et al., 2019). PGPR enhance nutrient availability, stimulate growth hormones, and suppress disease prevalence (Passari et al., 2018; Lyu et al., 2019; Rajaofera et al., 2020). Moreover, PGPR can suppress a broad range of pathogenic microbes including viruses, bacteria and fungi (Mazhar et al., 2016). Additionally, PGPR can improve plant health by acting as antagonists of pathogens using mechanisms such as solubilizing Fe and P, N fixation, or production of antibiotic compounds or hormones (Labuschagne et al., 2010; Ambreen et al., 2012; Beneduzi et al., 2012; Salomon et al., 2017). Disease suppression mechanisms include antibiosis, Induced Systemic Resistance (ISR), high affinity siderophore production, competition for nutrient and niches, and production of lytic enzymes (Rajiv et al., 2017; Salomon et al., 2017). The predominant genera of PGPR are *Pseudomonas* and *Bacillus* (Mazhar et al., 2016; Passari et al., 2018; Hashem et al., 2019). Members of genus *Bacillus* have been reported to be effective PGPR in a wide range of plants, and this genus is one of the principal PGPR groups known for their application as BCAs against several pathogenic fungi (Zhao et al., 2013; Leila et al., 2015; Ge et al., 2016; Mazhar et al., 2016; Hashem et al., 2019; Tiwari et al., 2019). Many *Bacillus* species are commonly isolated endophytes and are known to impart biological control against various diseases (Melnick et al., 2013). *Bacillus cereus* S42, isolated from *Nicotiana glauca* organs, suppressed *Fusarium* wilt in tomato (Aydi-Ben et al., 2016). Endophytic *B. subtilis* strain E1R displayed a biocontrol efficacy against wheat powdery mildew (Gao et al., 2015). *Bacillus methylotrophicus* strain NKG-1, isolated from the rhizosphere of a *Pinus koraiensis* in a dormant volcano in southern China, exhibited significant antifungal and pro-fertilization activities on tomato plants (Ge et al., 2016). *Bacillus subtilis* SQR9 showed potential to control *Fusarium* wilt in cucumber plants by root colonization (Cao et al., 2011). Es-soufi et al. (2020) showed that *B. amyloliquefaciens* Bc2 is a potent biocontrol agent against strawberry anthracnose. *B. amyloliquefaciens* Q-426 displayed a potential biocontrol ability against *Fusarium oxysporum* f. sp. *spinaciae* (Zhao et al., 2013). The importance of the composition of the rhizosphere microbiome on plant health and productivity has been increasingly recognized. Some studies revealed that the application of BCAs belonging to *Bacillus* spp. or *Trichoderma* spp. suppressed soil borne plant diseases and altered the composition of the rhizosphere microbial community in banana, cucumber, and potato (Shen et al., 2015; Han et al., 2019; Wang et al., 2019c).

Here, we showed *F. oxysporum* root rot disease incidence and severity in Chinese wolfberry was preventable by implementing a biocontrol strategy. With application of each of two bacterial BCAs (*B. amyloliquefaciens* strains HSB1 and FBZ42), we observed (1) inhibited growth of fungal mycelia along with enhanced plant growth, and (2) disease suppression through each BCA altering the bacterial composition of the wolfberry rhizosphere. Therefore, these strains offer potential as safe BCAs to protect Chinese wolfberry while also ensuring a good yield of plant material.

## Materials and Methods

### Fungal Pathogens and Bacterial Antagonists Used in This Study

We used five fungal pathogens, including *Fusarium oxysporum*, *F. solani*, *F. chlamydosporum*, *F. tricinctum* and *Alternaria alternata*, that were previously identified as root-rot-causing pathogens in wolfberry plants and they were preserved in glycerol at −80°C (Uwaremwe et al., 2021). The five fungi were cultivated at 25°C for 7 days on potato dextrose agar (PDA) that included 20% potato infusion, 2% dextrose, and 1.5% agar obtained from Qingdao Hope Bio-Technology Co., Ltd., in China. Afterward, a small block of mycelium agar was cut and placed into the center of a fresh PDA plate. One of the BCAs used in the current study was *Bacillus amyloliquefaciens* strain FBZ42, a commercial strain from the company ABiTEP GmbH that was donated by the *Bacillus* Genetic Stock Center (BGSC). This strain has shown both plant growth promotion and disease suppression potential for different plants (Kröber et al., 2014). The second bacterial BCA, HSB1, was an uncharacterized endophytic bacterium we isolated from wolfberry root tissues while conducting this study. Briefly, wolfberry roots were cleaned using 75% ethanol for 30 s and immediately transferred to 3% sodium hypochlorite for 5 min, and finally washed three times with sterile water. They were finally cut into small, thin blocks 0.5 cm × 0.5 cm, and placed on NA (Nutrient Agar) culture medium obtained from Qingdao Hope Bio-Technology Co., Ltd., in China, followed by incubation at 37°C. The FZB42 and HSB1 cells were grown and maintained in Luria Bertani liquid medium at 4°C for further experiment.

### *In vitro* Antifungal Assay

In the plate confrontation assay, a small block of agar covered with fungal mycelia was excised and placed onto the center of a fresh PDA plate and incubated for three additional days to ensure fungal colonization of the new plate. Afterward, FZB42 and HSB1 were added to Luria Bertani liquid medium (30 mL) and incubated for overnight at 37°C with shaking at 150 rpm. Then, 5 μl of either FZB42 or HSB1 cells were point inoculated on the PDA plate 2.5 cm away from the fungus. Their antifungal activity was evaluated by comparing fungal mycelium growth in the presence of each bacterial BCA, and plates that were inoculated with fungi alone (control plates), after 7 days of incubation at 25°C as reported in Cao et al. (2018).

Strain HSB1 was identified using 16S rRNA gene sequences as previously reported (Mignard and Flandrois, 2006; Janda and Abbott, 2007), after it had already displayed antifungal activity. The resulting 16S rRNA gene sequence was compared in a BLAST search to those in the National Library of Medicine (Bethesda, United States) database (Altschul et al., 1997). Phylogenetic analysis of HSB1 was performed using MEGA 5.5 (Kumar et al., 2008) and the relationships between HSB1 and other *Bacillus* sequences were analyzed using the neighbor-joining method (Saitou and Nei, 1987; Uwaremwe et al., 2021). Bootstrap values for the neighbor-joining tree were calculated for 1,000 replicates (Felsenstein, 1985; Uwaremwe et al., 2021).

### Effects of FZB42 and HSB1 on the Growth of Wolfberry Seedlings

In addition to assessing their antifungal abilities, FZB42 and HSB1 were tested twice for their potential to promote plant growth of Chinese wolfberry seedlings under laboratory conditions. The first assay, conducted from August to October 2019, compared the plant growth promotion potentials of HSB1 and FZB42. To prepare bacterial suspensions, each BCA was first added to Luria Bertani liquid medium (30 mL) and incubated for 12 h at 35°C with shaking at 150 rpm until the logarithmic growth phase was reached. Afterward, all cells were harvested by centrifugation at 5,000 rpm for 5 min, the supernatant was discarded, and the pellets were washed and resuspended in sterile distilled water to obtain initial bacterial population densities of 6 × 10^9^ (FZB42) and 4 × 10^9^ (HSB1) colony forming units (CFU) mL^–1^. Finally, sterilized distilled water was used to make the final suspension at 5 × 10^8^ CFU mL^–1^ for both HSB1 and FZB42 following the protocol of Cheng et al. (2019). *Lycium barbarum* “Ningxia N1” seeds obtained from the Institute of Plant Protection, Ningxia Academy of Agricultural and Forestry Sciences, China, were planted in three large plastic pots (40 cm × 60 cm) containing autoclaved pindstrup substrate (pH 5.5–6) obtained from market. Each pot was amended with 2 L of bacterial suspension, with FZB42 as treatment 1, HSB1 as treatment 2, and water control as treatment 3. When each plant had 3–4 leaves, seedlings were individually transferred to small plastic pots (3 seedlings/pot). In each pot was a mixture of autoclaved sand obtained from the Tengger desert in China (latitude 37°30′ to 40° north × longitude 102°20′ to 106° east), soil obtained from Gaolan county in Gansu province (latitude 36°05′–36°51′ north × longitude 103°32′–104°14′ east) and pindstrup substrate in a 1:1:1 ratio (vol/vol/vol), that was irrigated with 50 ml of each treatment suspension (FZB42, HSB1 or water). Root length (cm) and stem height (cm) were recorded for inoculated and non-inoculated seedlings at 60, 68, and 70 days after planting. The second assay was conducted from May through the end of June 2020, and involved HSB1 alone. *L. barbarum* “Ningxia N1” seeds were planted in a large plastic pot containing autoclaved pindstrup substrate as in the first assay, and the pot was amended with 2 L of water. When each had 3–4 leaves, seedlings were individually transferred to small plastic pots (1 seedling/pot) filled with a mixture of autoclaved sand, soil and pindstrup substrate (1:1:1 ratio, vol/vol/vol), and immediately irrigated with water. When seedlings reached 35 days of growth, HSB1 was inoculated at a concentration of 2 × 10^7^ CFU mL^–1^. Shoot weight (g) and root weight (g) were recorded for inoculated and non-inoculated seedlings every 7 days after inoculation. The two experiments were conducted according to a randomized complete design composed of three replicates for each treatment and water control.

### Biocontrol Experiment

In addition to the *in vitro* biocontrol experiments, and *in planta* biocontrol experiment was conducted whereby HSB1 and FZB42 were used as BCAs against a strain of *F. oxysporum* that was previously isolated from Chinese wolfberry and identified based on ITS and TEF (Genbank reference: MN959986 and MT811807) (Uwaremwe et al., 2021). *L. Barbarum* seedlings were obtained from seed germination. Briefly, seeds were surface sterilized with 75% ethanol for 30 s, sodium hypochlorite for 5 min, and finally washed with sterile distilled water five times. They were planted in two different large plastic pots containing autoclaved pindstrup substrate amended with tap water. All pots were kept in laboratory conditions at a temperature of 25°C, with humidity between 75 and 90%, and an alternating cycle of 16 h light / 8 h dark having a total light intensity of 800 μMol/m2/s. After 20 days, germinated seedlings were individually transplanted into different plug trays containing a mixture of soil, sand and pindstrup substrate in equal ratio (1:1:1 ratio, vol/vol/vol) autoclaved two times to ensure complete disinfection. At the 4–5 leaf developmental stage, individual seedlings were transplanted into their own large plastic pots containing autoclaved soil, sand and pindstrup substrate.

For inoculum preparation, HSB1 and FZB42 were prepared as previously described in the plant growth promotion experiment. To obtain the fungal inoculum, *F. oxysporum* was cultured on petri dishes containing PDA and incubated at 25°C for 10–15 days. A conidial suspension was prepared by pouring 30 mL of sterile distilled water into each of the petri dishes and dislodging spores with a sterile toothbrush. The initial concentration of conidia in the suspension was determined using a hemocytometer, and the final inoculum concentration was adjusted to 5 × 10^7^ conidia ml^–1^. Two methods of inoculation were used for this experiment. The first method involved inoculating seedlings with *F. oxysporum* 5 days before either FZB42 or HSB1 was introduced. The second inoculation procedure method involved inoculating FZB42 or HSB1 first, followed by a supplementary inoculation after 7 days to ensure their colonization as reported in Gadhave et al. (2018). After another 10 days, the BCA-treated seedlings were inoculated with *F. oxysporum*. In total, eight treatments were used: (1) CK, untreated seedlings (Control), (2) *F. oxysporum* alone, (3) FZB42 alone, (4) HSB1 alone, (5) *F. oxysporum* + FZB42, (6) *F. oxysporum* + HSB1, (7) FZB42 + *F. oxysporum* and (8) HSB1 + *F. oxysporum*. Seedlings were inoculated by pouring 40 ml of the prepared inoculum onto the soil surface. The control treatments consisted of an equivalent volume of sterile distilled water. This experiment was conducted according to a randomized complete design composed of three replicates for each treatment and water control.

Disease incidence (DI) was calculated according to the formula developed by Sharma and Kolte (1994) as the percentage of infected seedlings out of the total of all treated seedlings for each treatment, according to the following formula:

$$\mathrm{DI}=\frac{\mathrm{total}\,\mathrm{number}\,\mathrm{of}\,\mathrm{infected}\,\mathrm{seedlings}}{\mathrm{total}\,\mathrm{number}\,\mathrm{of}\,\mathrm{all}\,\mathrm{inoculated}\,\mathrm{seedlings}}\times 100$$

Disease severity (DS) of the foliage was evaluated using a rating scale from 0 to 4 as reported in Han et al. (2019). Based on different stages of root rot, 0 = seedlings with no symptoms, 1 = leaf yellowing, 2 = necrosis, 3 = wilting and 4 = leaf loss. The DS values were obtained from the averages of these scores. Biocontrol efficiency (BE) was calculated according to a formula by Wang et al. (2016) and Cheng et al. (2019) as follows:

$$\mathrm{BE}=\frac{\begin{array}{c} \mathrm{Mean}\,\mathrm{DI}\,\mathrm{in}\,\mathrm{pathogen}\,\mathrm{sole}\,\mathrm{treatment}\\ -\mathrm{Mean}\,\mathrm{DI}\,\mathrm{in}\,\mathrm{pathogen}+\mathrm{BCA}\,\mathrm{treatment} \end{array}}{\begin{array}{c} \mathrm{Mean}\,\mathrm{DI}\,\mathrm{in}\,\mathrm{pathogen}\,\mathrm{sole}\,\mathrm{treatment} \end{array}}\times 100$$

### Metagenomic Sequencing of Bacterial Community

Twenty-five days after *F. oxysporum* inoculation, rhizosphere soils for the three replicate pots were collected from all treatments (24 samples in total) and sieved (2 mm). Briefly, the roots were lightly shaken to remove loosely attached soil. The soil that was still tightly adhering to the roots was harvested as rhizosphere soil and frozen at −80°C for DNA extraction following the protocol of Wu et al. (2016). Total soil DNA was extracted using a Qiagen DNeasy PowerSoil Kit following all steps provided in the kit handbook^1^. Genomic DNA concentration and purity were measured using a Qubit fluorometer (Thermo Fisher Scientific, United States).

Bacterial community composition was assessed by sequencing the V1-V9 region of the 16S rRNA gene using PCR primers 27F (5′- AGRGTTTGATYNTGGCTCAG-3′) and 1492R (5′- TASGGHTACCTTGTTASGACTT-3′) as reported in Větrovsky and Baldrian (2013). PCR conditions were initiated at 95°C for 5 min, followed by 25 cycles of denaturation at 95°C for 30 s, annealing at 50°C for 30 s, and extension at 72°C for 1 min, followed by a final elongation at 72°C for 7 min, and then hold at 4°C. The PCR products were pooled and visualized on 1% agarose gels, purified using a MinElute PCR Purification Kit according to the manufacturer’s instructions, and quantified using QuantiFluorTM-ST (Promega, United States). High-throughput sequencing was carried out on the PacBio Sequel II platform (BioMarker Technologies Co., Ltd., China). The original subreads were first corrected to generate circular consensus sequences (CCS) (SMRT Link, version 8.0), and then Lima software (v1.7.0) was used to identify the CCS of different samples through their barcoded sequences, and UCHIME1 (version 8.1) was used to remove the chimera bodies for high quality CCS sequences. Using USEARCH 4 (version 10.22) with a cut-off of 97% similarity, the Operational Taxonomic Units (OTUs) were clustered and the taxonomic classifications were performed using RDP Classifier (Version 2.2, based on Bergey’s taxonomy) with the classification threshold set at 0.5.

Putative bacterial metagenomic functions were inferred using a phylogenetic investigation of communities by reconstruction with unobserved states (PICRUSt) on the 16S rRNA gene abundance data as reported by Langille et al. (2013). Using functions within the PICRUSt pipeline, the OTU-table was normalized and used for metagenome inferences involving the KEGG (Kyoto Encyclopedia of Genes and Genomes) orthologs (KOs). The predicted functions were then collapsed into hierarchical KEGG pathways using the “categorize by function” step in the PICRUSt pipeline as performed by Wilkinson et al. (2017).

### Statistical Analysis

Plant growth promotion data were analyzed using the analysis of variance (ANOVA) procedure of SAS 8.1 software (SAS Institute Inc., Cary, NC, United States). Differences between treatments were assessed at each time point by Fisher’s protected least significance difference (LSD) test at 0.05 levels. Kruskal–Wallis one-way analysis of variance by ranks was used for comparing DI and DS. The rarefaction curve, corresponding to observed OTUs at different sequencing depths, was examined using QIIME software to determine whether the depth was reasonable. Chao1 and abundance-based coverage estimator (ACE) indices were used to calculate the evenness of each sample (Bokulich et al., 2013), while the Shannon and Simpson indices were used to measure diversity (Hong et al., 2015). Beta diversity among samples was determined by principal component analysis (PCA) using R software^2^. Significant differences in bacterial community composition between paired samples were determined using the Metastats analysis and Mothur program which counted taxa in five classified levels (Lu et al., 2016). The BE values were not subjected to statistical analysis.

## Results

### Molecular Identification of Bacterial Strain HSB1 and Phylogenetic Analysis

**FIGURE 1:**
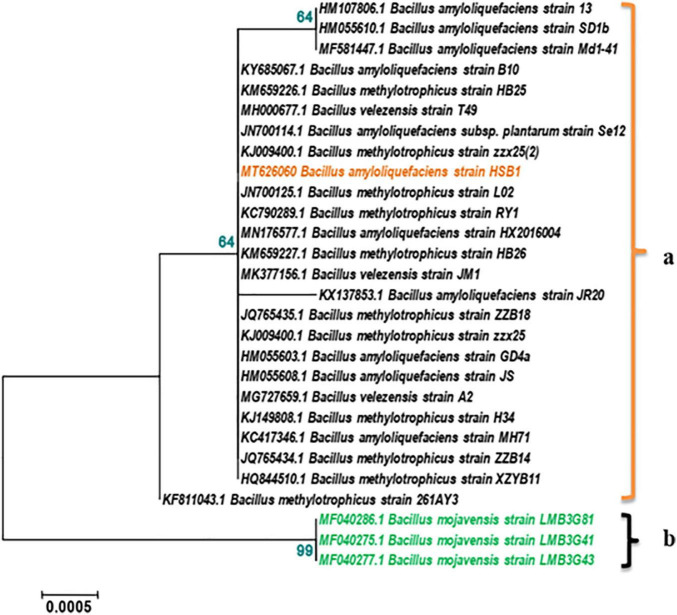
Phylogenetic tree based on 16S rRNA gene showing relationships between *Bacillus* strain HSB1 and other *Bacillus* strains/species derived from NCBI accessions. The tree was inferred using the neighbor-joining method and MEGA 5.5 software with 1000 bootstrap replicates (bootstrap values are shown next to nodes). The strain HSB1 clustered with *B. amyloliquefaciens*, *B. methylotrophicus*, and *B. velezensis* strains, **(a)**. *B. mojavensis* strains were used as out-group **(b)**.

16S rRNA was used to identify the genus and species of HSB1. This sequence has been accessioned to GenBank (MT626060). The BLAST result showed 99.93% homology with a strain of *Bacillus amyloliquefaciens.* The phylogenetic analysis showed HSB1 clustering with other *B. amyloliquefaciens*, *B. methylotrophicus*, and *B. velezensis* strains, which are known to share identity and a most recent common ancestor (Figure 1a). *B. mojavensis* strains were used as out-group (Figure 1b).

### *In vitro* Antifungal Assay

**FIGURE 2:**
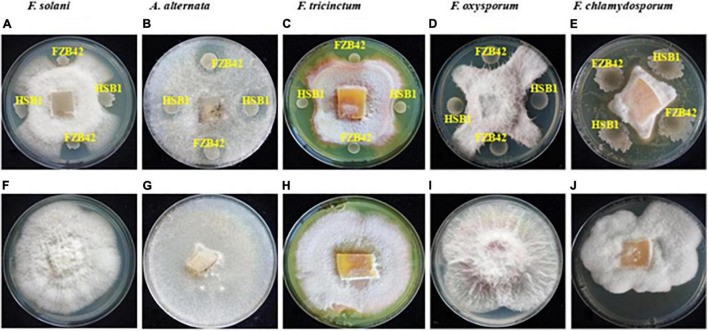
Plate assay showing antagonistic activity of both *B. amyloliquefaciens* FZB42 and HSB1 against each of five fungal pathogens of Chinese wolfberry: *F. solani*, *A. alternata*, *F. tricinctum*, *F. oxysporum* and *F. chlamydosporum* **(A–E)**. Panels **(F–J)** are the respective controls.

The potential of the two *B. amyloliquefaciens* strains, HSB1 and FBZ42, to inhibit the five root rot fungal pathogens (*F. oxysporum*, *F. solani*, *F. chlamydosporum*, *F. tricinctum* and *A. alternata*) was assessed using dual culture technique. The results showed that both HSB1 and FZB42 inhibited mycelial growth of all five fungal pathogens compared to the control (petri plates without bacterial served as control (Figure 2). All five fungal pathogens were inhibited (up to 100%). Due to its faster growth rate compared to the *Fusarium* strains tested, *A. alternata* was the first to show inhibition from exposure to our BCAs (Figures 2A–E).

### Effects of FZB42 and HSB1 on the Growth of Wolfberry Seedlings

**FIGURE 3:**
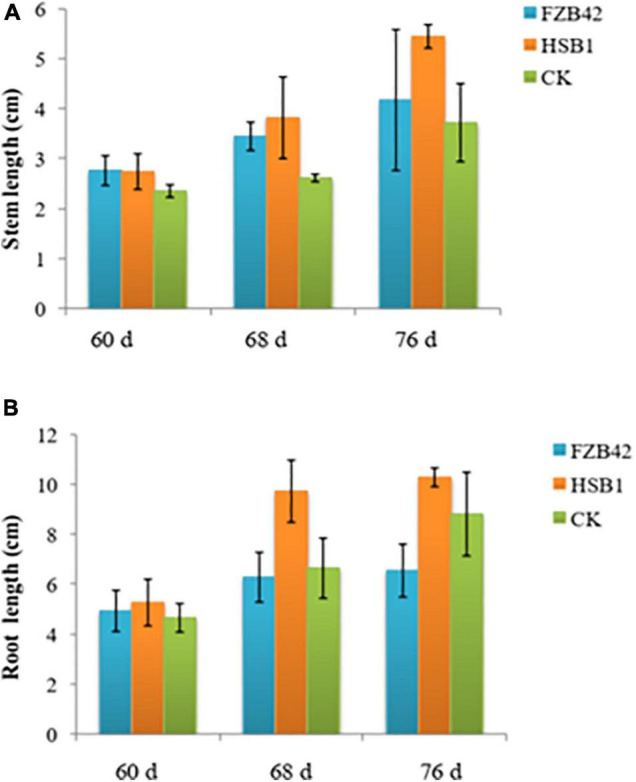
Bar graphs showing changes in stem length **(A)**, and root length **(B)** of wolfberry seedlings whose soil was treated with *Bacillus amyloliquefaciens* strains (FZB42 or HSB1) compared to a water control (CK). Measurement time points are based on growth at 60, 68, and 76 day. Error bars represent standard deviation of three replicates.

**FIGURE 4:**
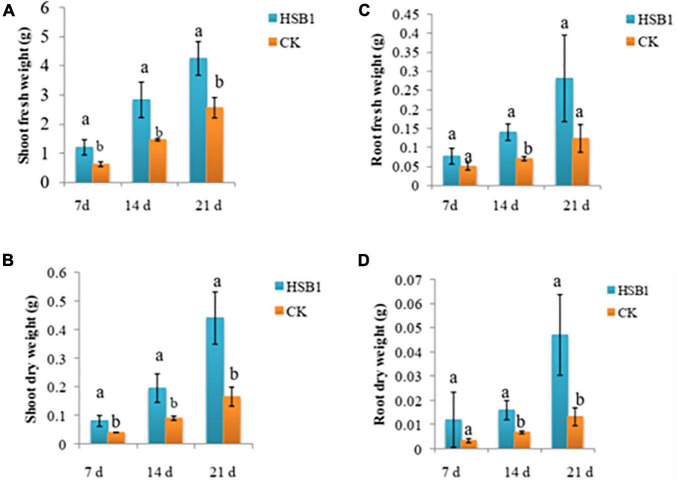
Bar graphs showing changes in shoot weight **(A,B)**, and root weight **(C,D)** of wolfberry seedlings whose soil was treated with *Bacillus amyloliquefaciens* strains (FZB42 or HSB1) compared to a water control (CK). Measurement time points are based on days after inoculation growth at 7, 14, and 21 days after inoculation. Error bars represent standard deviation of three replicates. Letters above each bar indicate significant differences from the control (CK) (*p* < 0.05).

This set of experiments showed that the two bacterial strains, FZB42 and HSB1, had different effects on the development of wolfberry seedlings. Both strains promoted plant growth in some way compared to the CK. In the first assay comparing HSB1 to FZB42, all seedlings were growing at same rate during first 60 days (Supplementary Figures 1A–C). In the days after, both FZB42 and HSB1 increased stem length compared to the water control. At 68 and 78 day time points, the greatest stem length was observed in HSB1-treated seedlings (Figure 3A). Measurements showed that both FZB42 and HSB1 increased root length in 60-day-old seedlings compared to the control. However, HSB1 had significantly increased the root length compared to both the FZB42 and control treatments, with FZB42-treated roots having shorter root length than even the water control treatment (Figure 3B). In the second assay, HSB1-treated seedlings were healthier and taller compared to control treatment (Supplementary Figures 1D,E). Results showed that HSB1 increased wolfberry seedlings shoot weight at the 7, 14, and 21 days post-inoculation time points compared to the control. There was a significant difference between HSB1 and control shoot weights (Figures 4A,B). HSB1also increased seedling root weight compared to control treatment. There was a significant difference between HSB1 and control at 14 and 21 day time after inoculation (Figures 4C,D).

### Biocontrol Experiment

**FIGURE 5:**
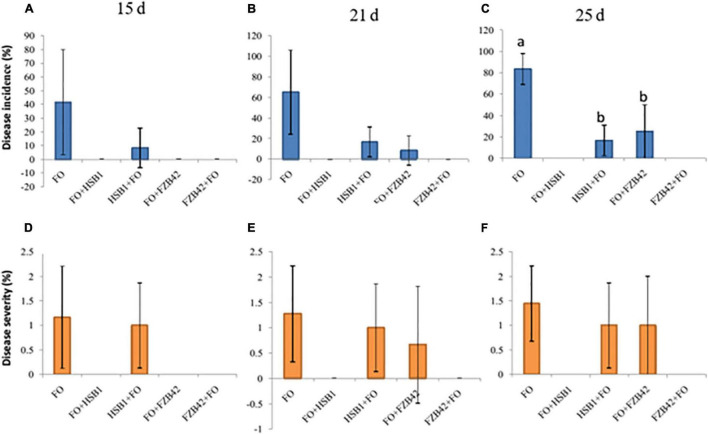
Bar charts showing disease incidence **(A–C)**, blue bars) and disease severity **(D–F)**, orange bars) for all treatments recorded at 15, 21, and 25 days time points. Error bars represent standard error of three replicateds. Letters above each bar indicate significant differences in disease incidence at 25 days between *F. oxysporum*, HSB1 + *F. oxysporum*, and *F. oxysporum* + FZB42, and treatments (*p* < 0.05).

As a baseline, DI and DS were assessed at 15, 21, and 25 day post-inoculation with *F. oxysporum* alone. The highest DI (42%) and DS (1.7) were recorded for day 15 (Table 1 and Figure 5). In the foliage, the disease usually was recognized by the yellowing, necrosis and wilting of leaves, followed by complete leaf loss (Table 1 and Supplementary Figure 2). At the beginning, infected seedlings were characterized by severe yellowing of leaves and the disease symptoms continually increased throughout the experiment (Supplementary Figures 2A,B and Table 1**).** Assessment conducted at 21 and 25 day after *F. oxysporum* inoculation revealed that the greatest respective DI (65 and 84%) and DS (1.28 and 1.45) values were also recorded in *F. oxysporum* alone (Figures 5B,C,E,F**)**. Unexpectedly, no DI and DS were recorded in treatments where *F. oxysporum* was inoculated before HSB1 (FO + HSB1) or where FZB42 was inoculated before *F. oxysporum* (FZB42 + FO) treatments throughout the experiment (BE = 100%); all seedlings had zero disease symptoms in the foliage (Supplementary Figures 2G,H). Additionally, control plants treated with water (CK), FZB42 alone, as well as HSB1 alone did not also show any disease symptoms (Supplementary Figures 2E,F,I). Brown lesions were observed on root surfaces of seedlings treated with FO alone or HSB1 + FO (Supplementary Figures 3A,C), whereas no lesions were observed on root surfaces of seedlings having the other treatments (Supplementary Figures 3B,D–H).

**TABLE 1:** Disease severity (DS) scores and averaged percentages of disease incidence (DI) observed with each treatment.

Treatment	DS 15 day	DS 21 day	DS 25 day	DI 15 day	DI 21 day	DI 25 day
FO	0	1	1	42%	65%	84%
	1	2	2			
	2	4	4			
	3					
HSB1 + FO	0	0	0	8%	17%	17%
	3	3	3			
FO + HSB1	0	0	0	0%	0%	0%
FO + FZB42	0	1	1	0%	8%	25%
	0	3	3			
FZB42 + FO	0	0	0	0%	0%	0%
HSB1	0	0	0	0%	0%	0%
FZB42	0	0	0	0%	0%	0%
CK	0	0	0	0%	0%	0%

*FO, HSB1 + FO, FO + HSB1, FO + FZB42, FZB42 + FO, HSB1, FZB42 and CK represent F. oxysporum, B. amyloliquefaciens HSB1 before F. oxysporum, F. oxysporum before B. amyloliquefaciens HSB1, F. oxysporum before B. amyloliquefaciens FZB42, B. amyloliquefaciens FZB41 before F. oxysporum, B. amyloliquefaciens HSB1, B. amyloliquefaciens FZB42 and control, respectively.*

### Metagenomic Sequencing of Bacterial Community

**FIGURE 6:**
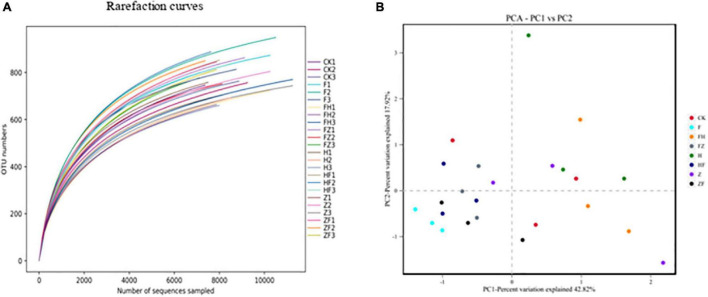
Rarefaction curves **(A)** showing the relationship between sequence number per sample and observed OTUs for different treatment replicates. The length of the curve reflects sequencing depth (i.e., a longer curve indicates a greater depth), while the smoothness reflects the effect of sequencing depth on sample diversity. Principal component analysis **(B)** based on the distance matrix calculated using the Bray-Curtis algorithm for soil samples collected from different treatments: CK, control; F, *F. oxysporum* alone; FH, *F. oxysporum* + HSB1; FZ, *F. oxysporum* + FZB42; H, HSB1 alone; HF, HSB1 + *F. oxysporum*; Z, FZB42 alone; ZF, FZB42 + *F. oxysporum*.

A total of 211,372 16S rRNA V1-V9 gene sequences were analyzed across 24 rhizosphere soil samples, with an average of 8,807 ± 959 sequences per soil sample (Supplementary Table 1). Based on a threshold of 97% shared nucleotide identity, these sequences were grouped by OTU, yielding 8,366, 8,259, 7,901, 7,484, 7,145, and 6,623 OTUs at the phylum, class, order, family, genus, and species levels, respectively (Table 2). The sequencing depth was analyzed to identify new taxa. A rarefaction curve analysis at 3% dissimilarity for the bacterial community revealed that the sharp of the curve was increasing and depth did not reach saturation, indicating that a greater sequencing depth was needed (Figure 6A). However, the data were sufficient for showing differences among the treatments and suggested that BCA application increased bacterial diversity. The richness indices (ACE and Chao1), and diversity indices (Shannon and Simpson) were further estimated and presented in Table 3. Chao1 and ACE indices were higher in HSB1 + FO and FZB42 + FO treatments compared to FO alone. The lowest values were recorded in HSB1 alone and FO + HSB1 treatments. In addition, Simpson index was higher in HSB1 alone and FO + HSB1 treatments compared to the FO alone treatment and CK, whereas the Shannon index was higher for FO + FZB42, FZB42 + FO, and HSB1 + FO treatments. PCA comparison of changes in soil bacterial community across different treatment groups showed that the first two principle components could explain 42.82 and 17.92% of the total variation. The bacterial communities differed according to treatment, indicating the effects of *F. oxysporum* and the two *Bacillus* strains on bacterial composition in the wolfberry rhizosphere (Figure 6B).

**TABLE 2:** OTU classification and corresponding numbers in with various treatments.

Treatment	Phylum	Class	Order	Family	Genus	Species
CK	7,990 ± 965	7,892 ± 969	7,537 ± 898	7,121 ± 807	6,698 ± 833	6,150 ± 828
F0	9,387 ± 817	9,271 ± 800	8,723 ± 738	8,006 ± 673	7,670 ± 696	7,006 ± 669
FO + HSB1	9,646 ± 1,636	9,588 ± 1,618	9,312 ± 1,539	9,099 ± 1,455	8,898 ± 1,425	8,444 ± 1,299
F0 + FBZ42	7,781 ± 951	7,654 ± 949	7,328 ± 945	6,979 ± 917	6,657 ± 793	6,110 ± 742
HSB1 + FZB42	7,590 ± 478	7,462 ± 474	7,141 ± 424	6,620 ± 399	6,227 ± 441	5,706 ± 359
FZB42 + FO	7,245 ± 487	7,101 ± 460	6,801 ± 522	6,307 ± 590	5,852 ± 667	5,365 ± 694
HSB1	7,957 ± 257	7,877 ± 261	7,515 ± 281	7,284 ± 253	7,067 ± 279	6,649 ± 252
FZB42	9,329 ± 1,959	9,225 ± 1,963	8,849 ± 1,891	8,455 ± 1,851	8,084 ± 1,791	7,554 ± 1,774

*CK, F, FH, FZ, HF, ZF, H and Z represent control, F. oxysporum, F. oxysporum + B. amyloliquefaciens HSB1, F. oxysporum + B. amyloliquefaciens FZB42, B. amyloliquefaciens HSB1 + F. oxysporum, B. amyloliquefaciens FZB42 + F. oxysporum, B. amyloliquefaciens HSB1, and B. amyloliquefaciens FZB42, respectively. Data were calculated from three replicates of each treatment and are shown as mean ± standard deviation.*

**TABLE 3:** The mean of the ACE, Chao1, and Simpson and Shannon indices of rhizosphere soil treatments with BCA and *F. oxysporum* at 97% similarity.

Treatment	Richness indices		Diversity indices	
	ACE	Chao1	Simpson	Shannon
CK	927.2 ± 18.07^cd^	919.9 ± 56.45^b^	0.0153 ± 0.0044b^cd^	5.37 ± 0.1558^bc^
F	1,013.5 ± 76.6^ab^	1,019 ± 60.94^a^	0.0078 ± 0.0023^d^	5.78 ± 0.1147^a^
FH	892 ± 45.55^cd^	897.2 ± 44.67^b^	0.022 ± 0.0021^ab^	5.04 ± 0.0769^d^
FZ	947.1 ± 68.22^bc^	934.2 ± 62.53^b^	0.0108 ± 0.0015^cd^	5.53 ± 0.0466^ab^
HF	1,038.8 ± 41.76^a^	1,043 ± 52.02^a^	0.0091 ± 0.0011^d^	5.66 ± 0.1240^a^
ZF	1,022 ± 8.690^ab^	1,025.1 ± 25.05^a^	0.0087 ± 0.0036^d^	5.69 ± 0.1936^a^
H	859.8 ± 33.79^d^	873.5 ± 45.18^b^	0.0271 ± 0.0075^a^	5.01 ± 0.1218^d^
Z	918.2 ± 38.95^cd^	917.2 ± 27.53^b^	0.0183 ± 0.0091^bc^	5.25 ± 0.2875^cd^

*CK, F, FH, FZ, HF, ZF, H and Z represent control, F. oxysporum, F. oxysporum + B. amyloliquefaciens HSB1, F. oxysporum + B. amyloliquefaciens FZB42, B. amyloliquefaciens HSB1 + F. oxysporum, B. amyloliquefaciens FZB42 + F. oxysporum, B. amyloliquefaciens HSB1, and B. amyloliquefaciens FZB42, respectively. Different letters in each column indicate statistically significant differences based on Duncan’s test (p < 0.05).*

**FIGURE 7:**
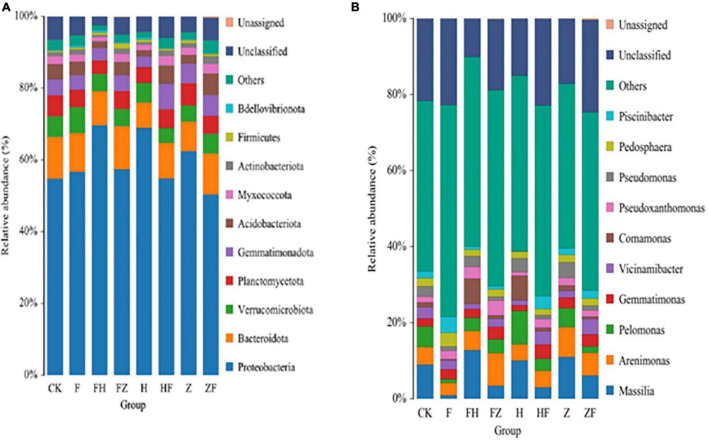
Stacked bar charts showing the relative abundances (in different colors) of the top 10 classified bacterial phyla **(A)** and genera **(B)** detected in soil samples subjected to different treatments: CK, control; F, *F. oxysporum* alone; FH, *F. oxysporum* + HSB1; FZ, *F. oxysporum* + FZB42; H, HSB1 alone; HF, HSB1 + *F. oxysporum*; Z, FZB42 alone; ZF, FZB42 + *F. oxysporum*. Relative abundance was based on the proportional frequencies of DNA sequences classified at the phylum and genus levels. Length of a color bar correlates with amount of abundance. Data were averaged from three replicates of each treatment.

The average number of microbial groups at phylum, class, order, family, genus and species levels were 24, 34, 73, 105, 192, and 239, respectively (Supplementary Table 2). All samples showed similar phylum and genus composition, but differed in terms of the relative abundances of other taxonomic groups (Figure 7). Of the 10 most abundant phyla across all samples, Proteobacteria and Bdellovibrionota were the least abundant. Although phylum Proteobacteria predominated, each treatment affected the relative abundance of this phylum in the overall rhizosphere bacterial composition (Figure 7A). The 10 most abundant genera included *Massilia* followed by *Arenimonas*, *Pelomonas*, *Gemmatimonas*, *Vicinamibacter*, *Comamonas*, *Pseudoxanthomonas*, *Pseudomonas*, *Pedosphaera* and *Piscinibacter* (Figure 7B). Consistent with our phylum level observations, relative abundances of *Massilia* differed by treatment (Figure 6B and Supplementary Table 3). Finally, LEfSe analysis was used to detect taxa with significantly different abundances between the FO *alone*, FO + HSB1, FO + FZB42, HSB1 alone, and FZB42 + FO treatments. The most differentially abundant bacterial taxa in the rhizosphere samples belonged to the Proteobacteria phylum (Supplementary Figure 4).

**FIGURE 8:**
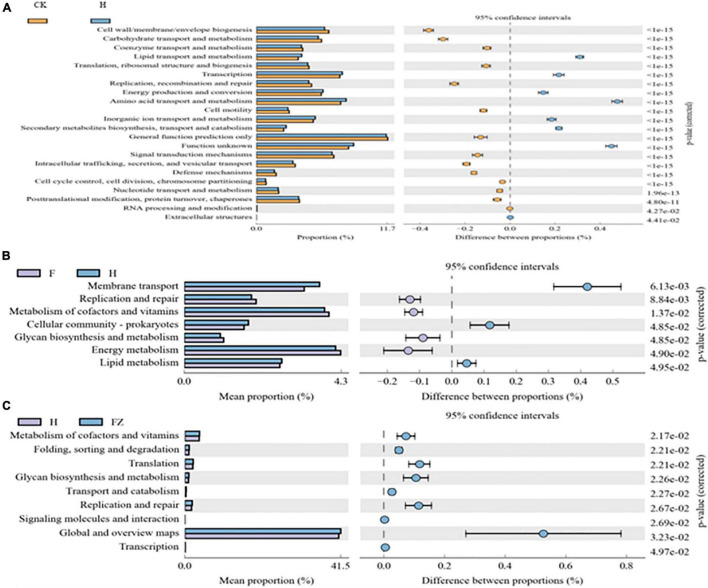
Metagenome comparisons, as predicted by PICRUSt, showing significant differences in the functionality of microbial genes detected in soil samples collected from selected treatments: CK, control; F, *F. oxysporum* alone, H, HSB1 alone, and FZ, *F. oxysporum* + FZB42. **(A)**: significant comparisons between CK and H treatments, **(B)**: significant comparisons between F and H treatments, and **(C)**: significant comparisons between H FZ treatments.

KEGG ortholog predictions were performed on the 16S rRNA data using PICRUSt. We conducted comparisons between different treatments, including CK vs. HSB1, *F. oxysporum* vs. HSB1, and HSB1 vs. *F. oxysporum* + FZB42 (Figure 7). Seven pathways related to lipid transport and metabolism, transcription, energy production and conversion, amino acid transport and metabolism, inorganic ion transport and metabolism, secondary metabolite biosynthesis, transport and catabolism, and “function unknown” were overrepresented in the HSB1 alone sample compared to the CK (Figure 8A). Comparison between *F. oxysporum* and HSB1 showed that pathways related to membrane transport, cellular community-prokaryotes, and lipid metabolism were overrepresented in the HSB1 alone sample (Figure 8B). In comparing HSB1 to FO + FZB42, pathways related to metabolism of cofactors and vitamins, translation, glycan biosynthesis and metabolism, replication and repair, global and overview maps were overrepresented in the FO + FZB42 sample (Figure 8C).

## Discussion

Currently, use of chemical pesticides represents one of the biggest ecological concerns, therefore biological control using beneficial microorganisms is considered a promising approach to manage soil borne diseases (Dhahira-Beevi and Qadri, 2010; Shahid and Khan, 2016; Singh et al., 2017; Alamri et al., 2019). Bacteria of the genus *Bacillus* are good candidates for use as BCAs (Falcäo et al., 2014; Chowdhury et al., 2015; Jiang et al., 2015). Therefore, a deep understanding of biocontrol mechanisms will help us to assess and to enhance the biological control of various diseases caused by soil borne pathogens in agriculture. This study determined the potential of two bacterial strains, belonging to the BCA genus *Bacillus*, to prevent root rot disease in wolfberry plants. Our results revealed that both HSB1 and FZB42 inhibited the mycelial growth of five fungal pathogens, which may be due to the production of antifungal secondary metabolites. This result is in line with previous studies showing the inhibition of fungal mycelial growth by different antagonistic bacterial strains. Falcäo et al. (2014) reported the antifungal activity of an endophytic bacterium, *B. subtilis* ALB629, that inhibited the mycelial growth of *F. solani* and *Colletotrichum gossypii*. Two other *Bacillus* strains, *B. subtilis* GM2 and *B. subtilis* GM5, isolated from the rhizosphere of potato roots have shown an ability to inhibit growth of different phytopathogenic fungi including *A. alternata* TP 712, *F. solani*, *F. oxysporum*, *F. redolens* and *Colletotrichum coccodes* 14raKK6 (Mardanova et al., 2017). *B. amyloliquefaciens* JDF35 was reported to inhibit the growth of *F. oxysporum* f. sp. *niveum* which causes wilt disease of watermelon (Zhao et al., 2017). The growth of *Colletotrichum gloeosporioides* and *F. oxysporum* was also reported to be inhibited by *Bacillus* species (BT42) isolated from the *Coffea arabica* rhizosphere (Kejela et al., 2016).

Beyond disease prevention, the application of HSB1 and FZB42 to wolfberry seeds resulted in seedlings with significantly stimulated the growth of shoots and/or roots. This finding is in agreement with previous studies that assessed plant-growth-promoting activity of various *Bacillus* strains in different plants (Falcäo et al., 2014; Yuan et al., 2015; Kejela et al., 2016). The addition of HSB1 and FZB42 before or after *F. oxysporum* inoculation significantly reduced DI and DS in wolfberry seedlings. Several studies reported the efficient application of *Bacillus* species as BCAs in the suppression of different pathogens causing diseases in plants (Zhang and Xue, 2010; Zhao et al., 2017; Kulimushi et al., 2018; Gautam et al., 2019).

Rhizosphere microbial communities play important roles in plant health and disease prevention (Dudenhöffer et al., 2016; Gu et al., 2016). An analysis based on richness indices (Chao1 and ACE, and Shannon), revealed that HSB1 and FZB42 altered the bacterial diversity of the rhizosphere in different ways. The inoculation of HSB1 5 days before *F. oxysporum* decreased Chao1 and ACE indices, while increasing the Simpson index, suggesting it negatively affected soil bacterial richness. However, inoculation of HSB1 and FZB42 17 days before *F. oxysporum* greatly increased the richness and Shannon indices, indicating that these BCAs positively affected soil bacterial richness. The abundances of rhizosphere microorganisms can be perturbed by biotic and abiotic factors (Ding et al., 2014; Huang et al., 2017). For instance, the introduction of additional bacteria and fungi can change native community structure (Karpouzas et al., 2011). The analysis of bacterial community composition and structure revealed that all treatments harbored structurally distinct taxa. Previous studies reported Proteobacteria, Acidobacteria, Actinobacteria, Bacteroidetes and Gemmatimonadetes as the predominant phyla in most rhizosphere soils (Zhu et al., 2013; Huang et al., 2017). Proteobacteria is the most abundant phylum in various soils (Shang et al., 2016; Wan et al., 2017; Han et al., 2019). This was also the predominant phylum represented in our study. Bacteria belonging to Proteobacteria generally expand faster by absorbing root-associated carbon substrates. Consequently, their abundance is positively proportional with carbon availability (Cleveland et al., 2006; Fierer et al., 2007). We also found that the relative abundance of Proteobacteria was elevated in rhizosphere soils in which *F. oxysporum* was inoculated first, more significantly in the FO + HSB1 treatment compared to FO alone. A similar result was obtained by Wan et al. (2017) after first inoculating FO, followed by BCA inoculation 7 days later. Assessment of DI showed asymptomatic seedlings in the FO + HSB1 treatment, indicating that the increased abundance of Proteobacteria may be associated with seedling protection and growth enhancement. Bacteroidota was the second-most abundant phylum in this study, a result confirmed by Han et al. (2019). The relative abundance of Actinobacteriota was found reduced in the FO alone treatment compared to HSB1 + FO, FZB42 + FO and FO + FZB42 treatments. This finding is consistent with previous reports, in which the Actinobacteriota phylum was found to be associated with disease suppression, due to its higher abundance in many disease-suppressive soils (Hunter et al., 2006; Fu et al., 2016). Proteobacteria was also reported to be highly abundant in disease-suppressive soil, because this group is known to produce high levels of secondary metabolites that inhibit pathogens (Mendes et al., 2011; Rosenzweig et al., 2012). Hence, a great abundance of Proteobacteria and Actinobacteria in soils should correlate with higher disease suppression ability.

*Massilia* is a rhizosphere-inhabiting and root-colonizing bacterium that associates with various plant species (Mendes et al., 2011; Rosenzweig et al., 2012). In our study, it showed to be most abundant in all treatments although its relative abundance varied markedly. It was found to be highly represented in FO + HSB1 treatment (DI and DS = 0) compared to *F. oxysporum* alone. A reduction in the relative abundance of *Massilia* was also reported in *F. oxysporum* treatment (Wan et al., 2017). Cretoiu et al. (2013) reported that genus *Massilia* suppresses soil borne diseases, thus the inoculation of HSB1 and FZB42 may synergistically protect wolfberry seedlings from root rot. *Arenimonas*, the second-most abundant genus, was highly represented in the FO + FZB42 treatment, and less abundant in the FO alone treatment. Jeong et al. (2016) reported a novel species of the genus *Arenimonas*, isolated from estuary sediment, to be oxidase- and catalase-positive. Genus *Pseudomonas* was highly represented in FZB42 alone and HSB1 alone treatments. This genus is known to display an ability to suppress different pathogens through various mechanisms such as production of antimicrobial compounds, induction of systemic resistance, promotion of plant growth, production of siderophores, and sequestration of nutrients (Li et al., 2012; Kong et al., 2016; Ma et al., 2017). Functional predictions associated with a microbiome are key to understanding the way the microbial community interacts with its environment. The recently-developed PICRUSt program was previously shown to be effective at obtaining functional predictions from 16S rRNA taxonomic data (Langille et al., 2013). Therefore, with the help of PICRUSt, we were able to gain functional insights into the bacterial community within the wolfberry rhizosphere. We noticed that HSB1 and FZB42 application significantly affected the function of the rhizosphere bacteria supporting a result reported by Han et al. (2019). Mendes et al. (2014) reported that the function of membrane transport may be associated with plant growth promotion and nutrition in the soybean rhizosphere. Plant growth promoting rhizobacteria are known to produce secondary metabolites antagonistic to various soil borne pathogens. In this study, introduction of HSB1 and FZB42 increased biosynthesis of secondary metabolites. A study by Berg et al. (2007) demonstrated that energy metabolism and signal transduction may improve resistance to *Fusarium* wilt in banana. Our findings will help us to develop an environmentally friendly and potent method to combat different pathogens responsible for root rot disease in Chinese wolfberry.

## Conclusion

The endophytic bacterium, *B. amyloliquefaciens* HSB1, isolated from wolfberry root tissues and characterized in this study, as well as *B. amyloliquefaciens* FZB42, efficiently inhibited several root rot pathogens of Chinese wolfberry plants. Their abilities to concomitantly enhance plant growth and the presence of other beneficial microbes showed their potential as suitable BCAs. However, a field study involving these potential BCAs is needed to support our laboratory findings.

## Data Availability Statement

The datasets presented in this study can be found in online repositories. The names of the repository/repositories and accession number(s) can be found in the article/Supplementary Material.

## Author Contributions

CU: experiments, data processing, interpretation, writing, and submitting. LY, YW, YT, XZ, YL, QZ, and YZ: experiments. RW: project coordination and supervising. All authors contributed to the article and approved the submitted version.

## Conflict of Interest

The authors declare that the research was conducted in the absence of any commercial or financial relationships that could be construed as a potential conflict of interest.

## Publisher’s Note

All claims expressed in this article are solely those of the authors and do not necessarily represent those of their affiliated organizations, or those of the publisher, the editors and the reviewers. Any product that may be evaluated in this article, or claim that may be made by its manufacturer, is not guaranteed or endorsed by the publisher.

## Footnotes

1
www.qiagen.com

2
https://www.rproject.org/
