# Supplementary material for: An Endophytic Strain of Bacillus amyloliquefaciens Suppresses Fusarium oxysporum Infection of Chinese Wolfberry by Altering Its Rhizosphere Bacterial Community
Source: Front Microbiol. 2022 Jan 5;12:782523. doi: 10.3389/fmicb.2021.782523 (PMC8767019; doi:10.3389/fmicb.2021.782523)
Supplement: Supplementary file 1 [file Data_Sheet_1.PDF]

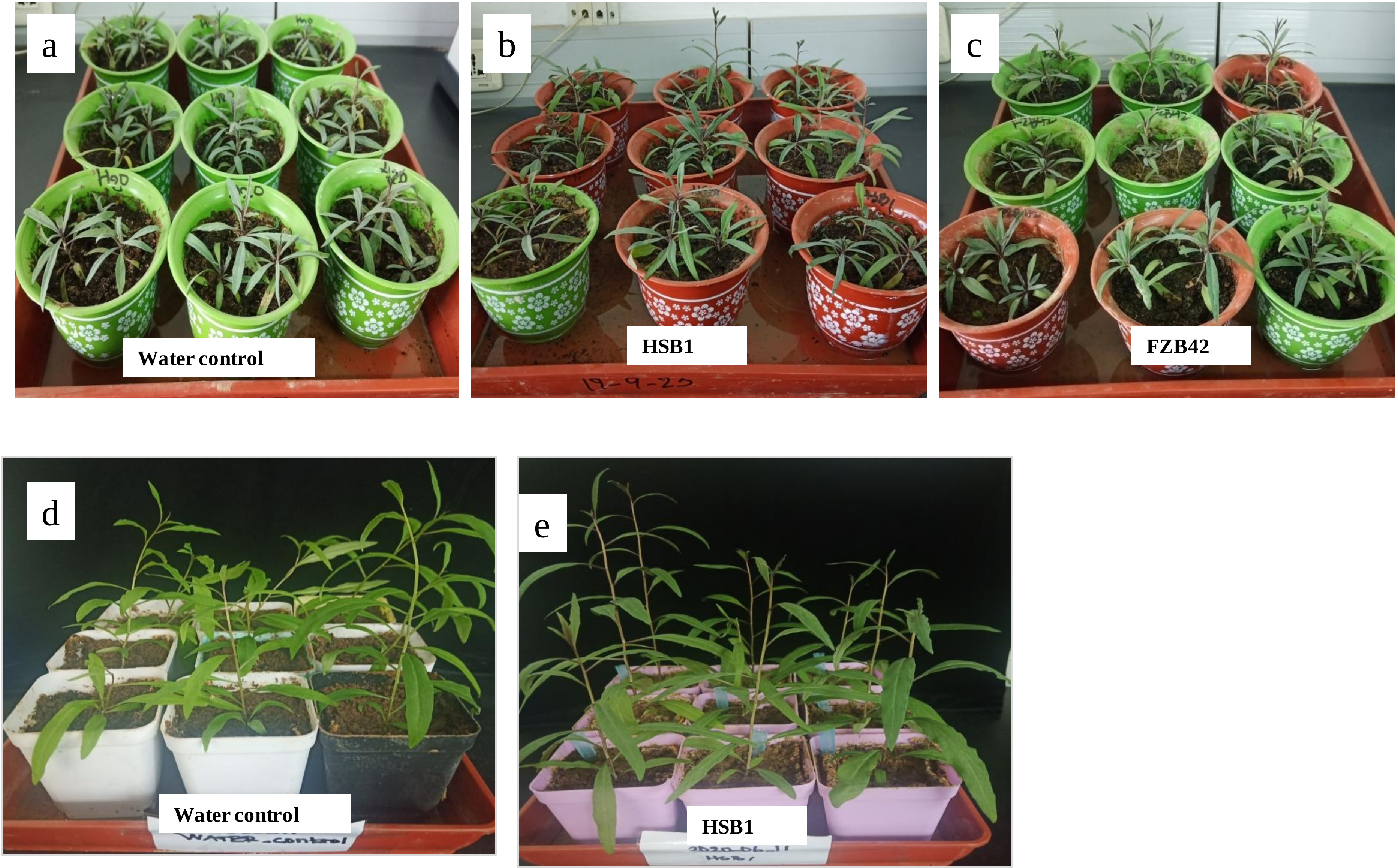

Figure S1

Figure S1. Growth promotion experiment. Assay 1: Water control seedlings (a), HSB1 treated seedlings (b) and FZB42 treated seedlings (c); Assay 2 : Water control seedlings (d) and HSB1 treated seedlings (f).

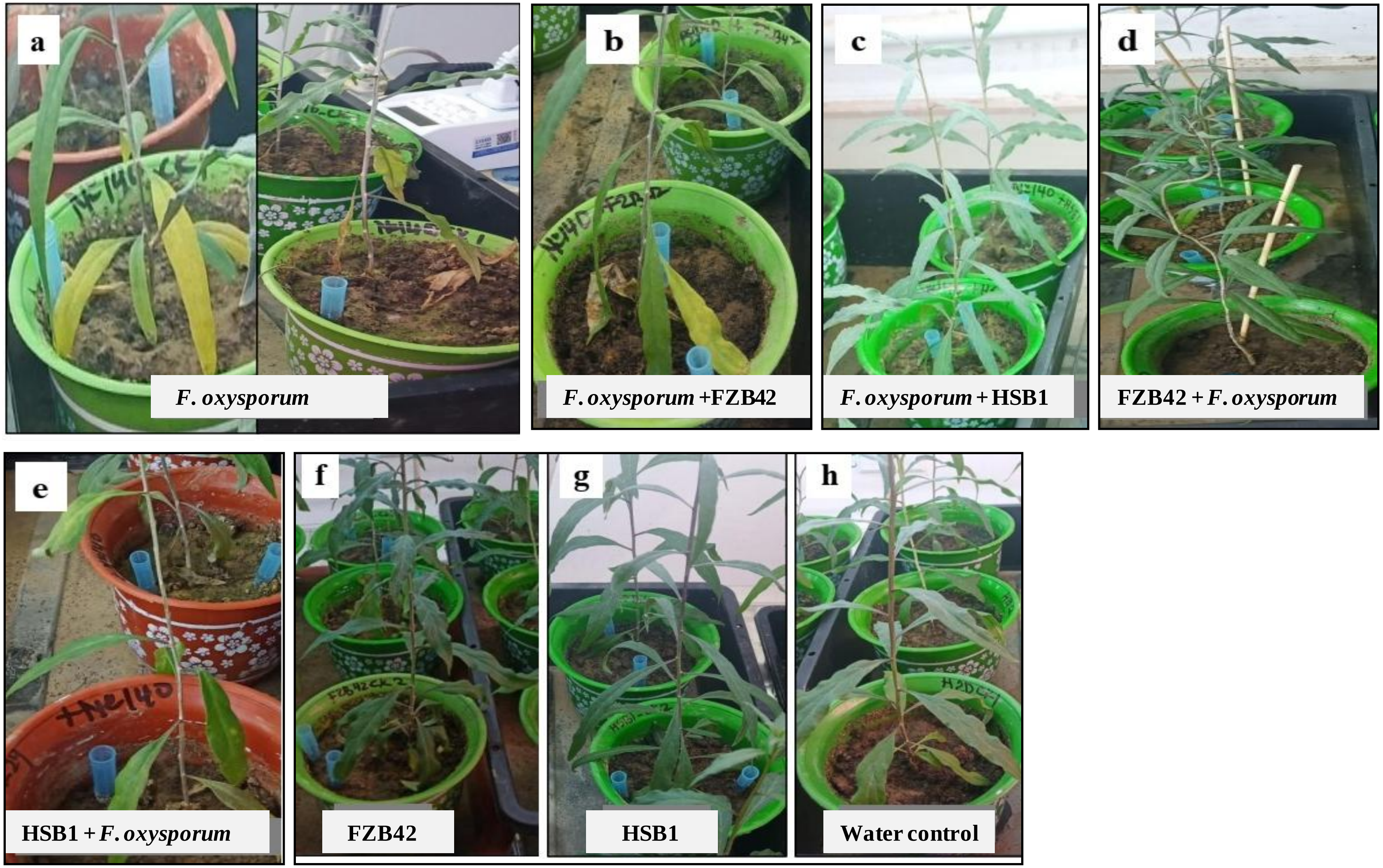
a
F. oxysporum
b
F. oxysporum +FZB42
c
F. oxysporum + HSB1
d
FZB42 + F. oxysporum
e
HSB1 + F. oxysporum
f
FZB42
g
HSB1
h
Water control

Figure S2

Figure S2. Images of wolfberry seedlings germinated seeds that had been subjected to eight different treatments during the biocontrol experiments. Treatment with *F. oxysporum* alone resulted in severe leaf yellowing and loss (a). Treatment with *F. oxysporum* + FZB42 resulted in 1-2 leaves turning yellow (b). Treatment with *F. oxysporum* + HSB1 resulted in no observable symptoms (c). Treatment with FZB42 + *F. oxysporum* resulted in no observable symptoms (d). Treatment with HSB1 + *F. oxysporum* resulted in weak leaves that were yellowing (e). Treatment with FZB42 alone resulted in no observable symptoms (f). Treatment with HSB1 alone resulted in no observable symptoms (g). Control treatment with water alone resulted in no observable symptoms (h).

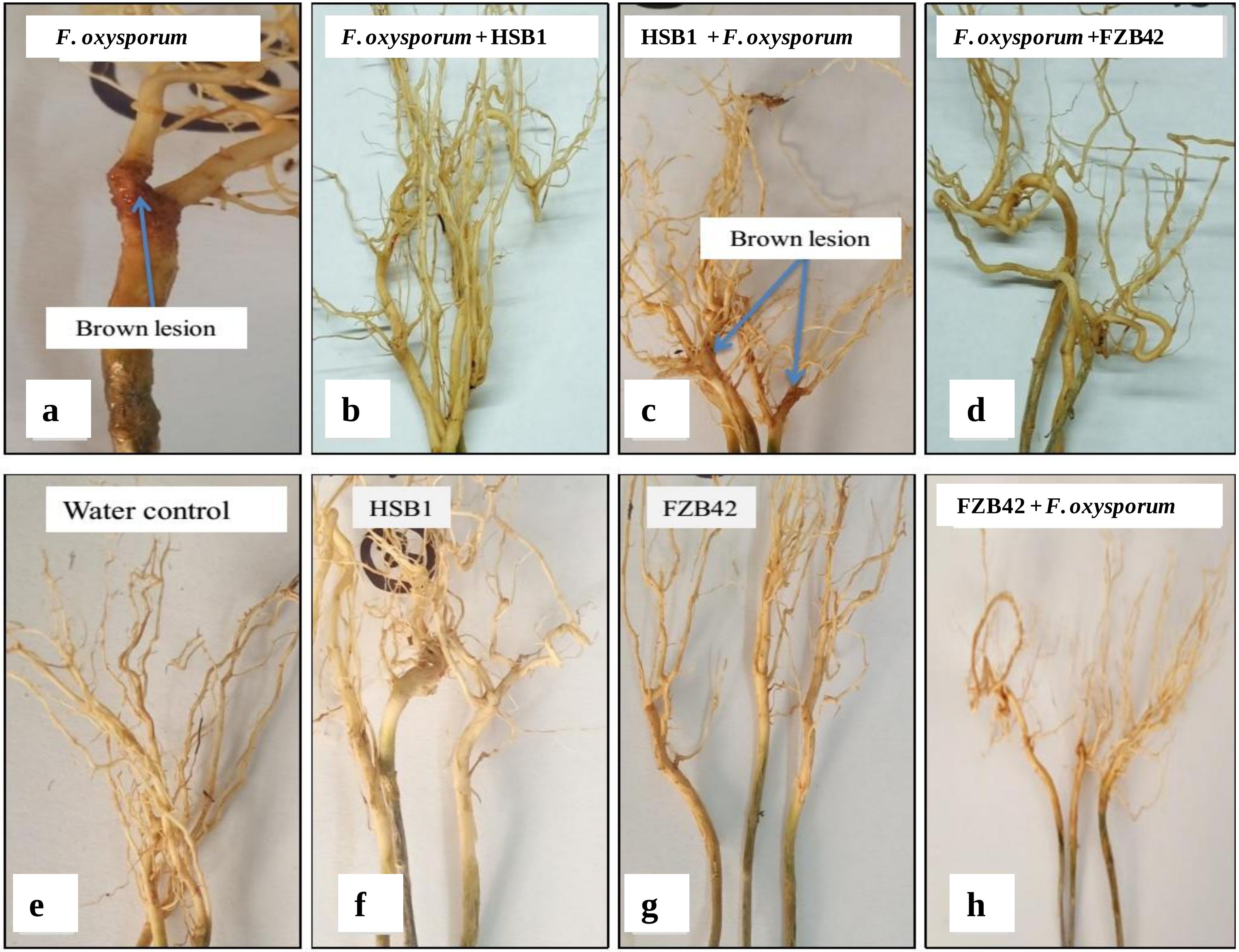
F. oxysporum
Brown lesion
a
F. oxysporum + HSB1
b
HSB1 + F. oxysporum
Brown lesion
c
F. oxysporum +FZB42
d
Water control
e
HSB1
f
FZB42
g
FZB42 + F. oxysporum
h

Figure S3

Figure S3. Images of wolfberry seedling roots after treatment with *F. oxysporum* alone (a); *F. oxysporum* + HSB1 (b); HSB1 + *F. oxysporum* (c); *F. oxysporum* + FZB42 (d); water control (e); HSB1 alone (f); FZB42 alone (g); and FZB42 + *F. oxysporum* (h).

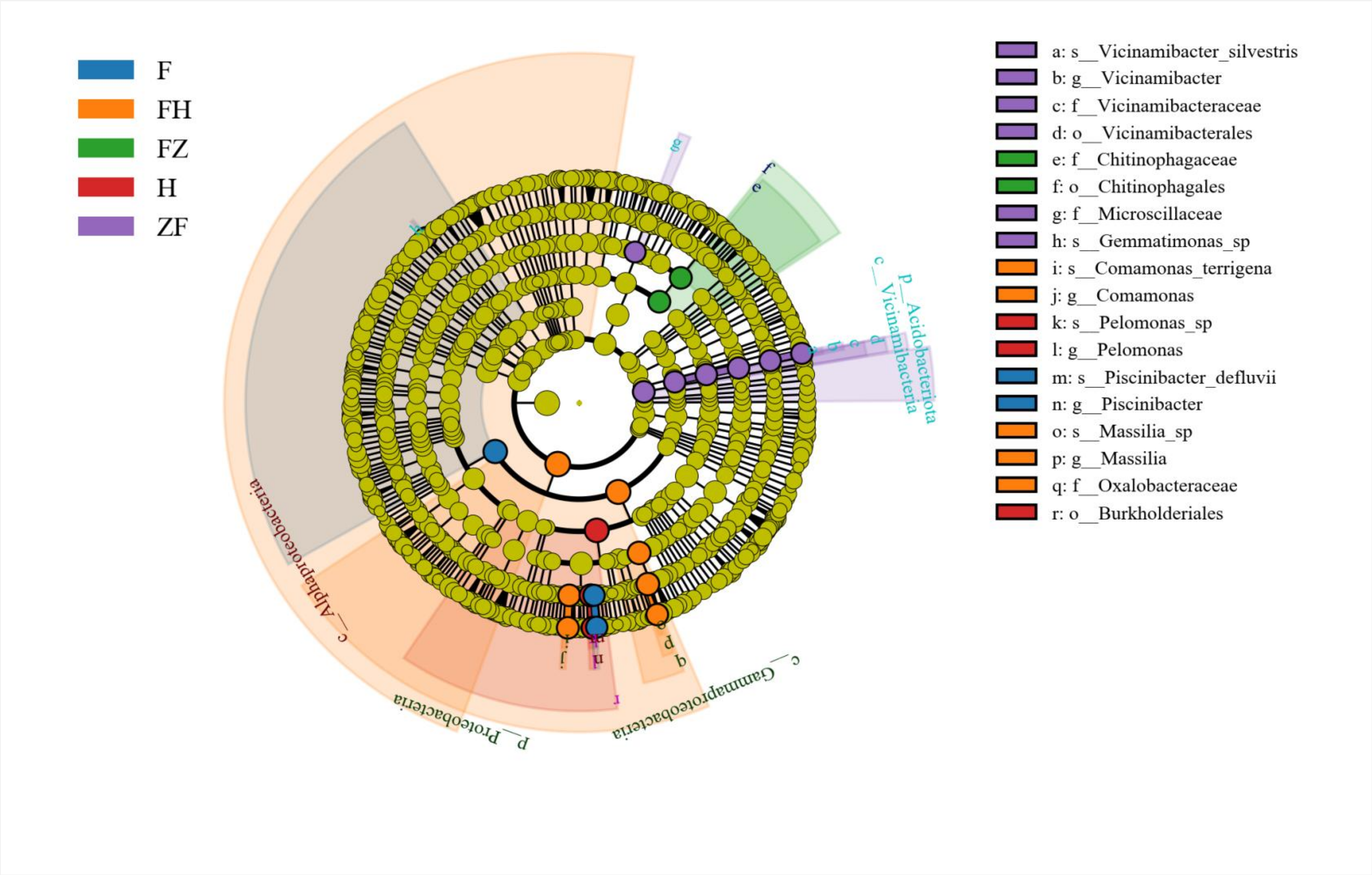

F
FH
FZ
H
ZF
a: s__Vicinamibacter_silvestris
b: g__Vicinamibacter
c: f__Vicinamibacteraceae
d: o__Vicinamibacterales
e: f__Chitinophagaceae
f: o__Chitinophagales
g: f__Microscillaceae
h: s__Gemmatimonas_sp
i: s__Comamonas_terrigena
j: g__Comamonas
k: s__Pelomonas_sp
l: g__Pelomonas
m: s__Piscinibacter_defluvii
n: g__Piscinibacter
o: s__Massilia_sp
p: g__Massilia
q: f__Oxalobacteraceae
r: o__Burkholderiales
p__Acidobacteriota
c__Vicinamibacteria
c__Alphaproteobacteria
p__Proteobacteria
c__Gammaproteobacteria

Figure S4

Figure S4. Cladogram comparing treatment-based variation in bacterial communities using LEfSe analysis. The legend to the left of the cladogram color-codes each treatment: F= *F. oxysporum* alone (blue); FH = *F. oxysporum* + HSB1 (orange); FZ = *F. oxysporum* + FZB42 (green); H =HSB1 alone (red); and ZF = FZB42 + *F. oxysporum* (purple). The legend to the right of the cladogram shows the predominant orders (o), families (f), genera (g) and species (s) present in the wolfberry rhizospheres for each treatment.
