# Supplementary material for: An Endophytic Strain of Bacillus amyloliquefaciens Suppresses Fusarium oxysporum Infection of Chinese Wolfberry by Altering Its Rhizosphere Bacterial Community
Source: Front Microbiol. 2022 Jan 5;12:782523. doi: 10.3389/fmicb.2021.782523 (PMC8767019; doi:10.3389/fmicb.2021.782523)
Supplement: Supplementary file 2 [file Table_1.DOCX]

**Table S1.** Gene counts for 16S rRNA sequences from each of three soil sample replicates across different treatments.

| Treatment | Bacterial 16S rRNA gene counts |
| --- | --- |
|  |  |
| CK1 | 7,376 |
| CK2 | 9,283 |
| CK3 | 8,989 |
| F1 | 10,318 |
| F2 | 10,597 |
| F3 | 8,839 |
| FH1 | 10,447 |
| FH2 | 7,976 |
| FH3 | 11,251 |
| FZ1 | 9,200 |
| FZ2 | 8,167 |
| FZ3 | 7,220 |
| H1 | 8,230 |
| H2 | 8,611 |
| H3 | 8,094 |
| HF1 | 8,059 |
| HF2 | 7,639 |
| HF3 | 8,513 |
| Z1 | 7,547 |
| Z2 | 10,302 |
| Z3 | 11,419 |
| ZF1 | 7,881 |
| ZF2 | 7,420 |
| ZF3 | 7,994 |
| Mean | 8,807± 959 |
| Total | 211,372 |

Treatments include a water control (CK), *F. oxysporum* only (F), *F. oxysporum* + HSB1 (FH), *F. oxysporum* + FZB42 (FZ), HSB1 only (H), HSB1 + *F. oxysporum* (HF), FZB42 alone (Z), and FZB42 + *F. oxysporum* (ZH).

| **Table S2.** Numbers of microbial groups representing each classification level based on different soil treatments | | | | |  |  |
| --- | --- | --- | --- | --- | --- | --- |
|  |  |  |  |  |  |  |
| **Sample** | **Phylum** | **Class** | **Order** | **Family** | **Genus** | **Species** |
| CK | 25±1.0000 | 34±1.5275 | 74±3.2146 | 106±4.0415 | 188±7.9373 | 234±13.077 |
| F | 25±2.0817 | 36±2.0817 | 77±4.0000 | 108±4.0415 | 198±11.930 | 242±17.349 |
| FH | 23±1.0000 | 31±2.0817 | 71±2.6458 | 101±2.3094 | 193±10.536 | 243±12.662 |
| FZ | 23±1.5275 | 34±2.3094 | 74±1.5275 | 104±1.1547 | 193±7.5498 | 241±4.0415 |
| H | 24±1.1547 | 32±1.0000 | 68±2.6458 | 100±3.2146 | 188±2.3094 | 236±3.5119 |
| HF | 26±1.5275 | 34±0.5774 | 75±1.1547 | 108±1.5275 | 188±4.5092 | 232±2.6458 |
| Z | 24±0.5774 | 32±1.5275 | 69±0.5774 | 101±1.0000 | 189±6.6583 | 241±14.189 |
| ZF | 25±0.5774 | 35±0.5774 | 76±1.0000 | 109±2.3094 | 197±10.599 | 241±17.243 |

Treatments include a water control (CK), *F. oxysporum* only (F), *F. oxysporum* + HSB1 (FH), *F. oxysporum* + FZB42 (FZ), HSB1 only (H), HSB1 + *F. oxysporum* (HF), FZB42 alone (Z), and FZB42 + *F. oxysporum* (ZH). Data were averaged from three replicates for each treatment and are shown ± standard deviation

**Table S3.** The 10 most abundant genera (%) observed with each of three soil sample replicates across different treatments

| **Treatment** | ***Massilia*** | ***Arenimonas*** | ***Pelomonas*** | ***Gemmatimonas*** | ***Vicinamibacter*** |
| --- | --- | --- | --- | --- | --- |
| CK1 | 12.70 | 5.27 | 2.19 | 2.48 | 2.63 |
| CK2 | 12.94 | 4.28 | 6.49 | 2.27 | 1.90 |
| CK3 | 1.88 | 4.43 | 6.89 | 1.66 | 4.26 |
| F1 | 0.40 | 3.17 | 1.57 | 2.54 | 2.29 |
| F2 | 1.04 | 2.65 | 0.80 | 2.43 | 2.63 |
| F3 | 1.28 | 4.23 | 0.36 | 2.83 | 2.34 |
| FH1 | 7.80 | 4.36 | 5.70 | 4.55 | 1.46 |
| FH2 | 17.13 | 2.39 | 2.43 | 1.22 | 1.68 |
| FH3 | 14.35 | 7.59 | 2.23 | 0.90 | 1.00 |
| FZ1 | 2.85 | 9.94 | 5.35 | 3.97 | 2.67 |
| FZ2 | 2.93 | 3.78 | 3.36 | 1.81 | 1.59 |
| FZ3 | 4.57 | 12.56 | 1.63 | 3.86 | 2.24 |
| H1 | 8.56 | 5.40 | 4.49 | 1.28 | 1.55 |
| H2 | 16.76 | 4.90 | 5.81 | 1.59 | 1.33 |
| H3 | 4.54 | 2.38 | 16.23 | 1.58 | 1.14 |
| HF1 | 1.11 | 4.67 | 5.47 | 3.73 | 4.05 |
| HF2 | 2.84 | 3.87 | 1.55 | 3.99 | 3.58 |
| HF3 | 4.91 | 4.59 | 2.54 | 3.25 | 2.97 |
| Z1 | 5.86 | 5.43 | 4.84 | 3.11 | 2.01 |
| Z2 | 8.08 | 7.00 | 6.99 | 3.62 | 2.02 |
| Z3 | 16.91 | 10.31 | 3.31 | 1.63 | 1.49 |
| ZF1 | 4.39 | 7.21 | 1.64 | 2.69 | 3.97 |
| ZF2 | 2.33 | 3.00 | 2.08 | 3.66 | 4.02 |
| ZF3 | 11.43 | 7.44 | 1.27 | 3.25 | 4.06 |

**Table S3**. Continued

| **Treatment** | ***Comamonas*** | ***Pseudoxanthomonas*** | ***Pseudomonas*** | ***Pedosphaera*** | ***Piscinibacter*** |
| --- | --- | --- | --- | --- | --- |
| CK1 | 0.88 | 2.26 | 4.12 | 1.93 | 0.122 |
| CK2 | 2.41 | 1.18 | 3.60 | 2.02 | 3.30 |
| CK3 | 0.34 | 1.33 | 0.57 | 2.43 | 1.61 |
| F1 | 0.34 | 3.39 | 0.37 | 4.32 | 6.77 |
| F2 | 0.22 | 1.76 | 1.20 | 3.39 | 3.08 |
| F3 | 0.10 | 1.53 | 1.78 | 2.98 | 2.62 |
| FH1 | 8.59 | 6.45 | 3.34 | 0.44 | 0.91 |
| FH2 | 6.61 | 2.26 | 2.41 | 1.44 | 0.35 |
| FH3 | 4.84 | 0.58 | 2.42 | 3.23 | 1.07 |
| FZ1 | 1.01 | 4.76 | 0.5 | 2.04 | 0.91 |
| FZ2 | 0.95 | 2.03 | 1.75 | 1.82 | 0.94 |
| FZ3 | 0.74 | 4.79 | 0.91 | 1.71 | 0.84 |
| H1 | 7.58 | 0.55 | 5.01 | 2.11 | 0 |
| H2 | 6.68 | 0.41 | 4.87 | 1.19 | 0.44 |
| H3 | 4.92 | 2.06 | 0.88 | 1.79 | 0.04 |
| HF1 | 0.81 | 2.38 | 0.34 | 1.31 | 3.11 |
| HF2 | 0.60 | 1.79 | 1.43 | 2.12 | 5.85 |
| HF3 | 1.37 | 2.57 | 1.31 | 1.35 | 1.58 |
| Z1 | 1.59 | 2.58 | 2.09 | 2.33 | 1.32 |
| Z2 | 1.51 | 2.44 | 3.85 | 1.82 | 2.72 |
| Z3 | 1.16 | 1.27 | 5.64 | 1.71 | 1.10 |
| ZF1 | 0.97 | 1.35 | 1.28 | 1.00 | 3.67 |
| ZF2 | 0.431 | 1.71 | 0.95 | 2.06 | 2.08 |
| ZF3 | 0.51 | 1.87 | 1.43 | 2.62 | 0.53 |

Treatments include a water control (CK), *F. oxysporum* only (F), *F. oxysporum* + HSB1 (FH), *F. oxysporum* + FZB42 (FZ), HSB1 only (H), HSB1 + *F. oxysporum* (HF), FZB42 alone (Z), and FZB42 + *F. oxysporum* (ZH).
